# FDA_YOLOv8: refined small object detection in unmanned aerial vehicle imagery

**DOI:** 10.1038/s41598-026-51902-3

**Published:** 2026-05-25

**Authors:** Weihong Chen, Qianjie Deng, Ziyan Xiang, Qianwen Cao, Jiwu Peng

**Affiliations:** 1 College of Computer and Artificial Intelligence, Hunan University of Finance and Economics, Changsha, 410125 China; 2 State Key Laboratory of Geological Processes and Mineral Resources, China University of Geosciences, Beijing, 100083 China

**Keywords:** Computational biology and bioinformatics, Engineering, Mathematics and computing

## Abstract

Small object detection (SOD) is essential for security monitoring in unmanned aerial vehicle (UAV) imagery. However, the inherently low effective resolution, weak semantic representation, and cluttered background of small objects pose significant challenges. Although deep learning methods have been widely applied to extract multi-scale features from UAV images, their performance remains limited by the small size of objects and complex scene variations. In addition, the constrained computational resources of UAV platforms make achieving both accuracy and efficiency in SOD even more challenging. In this study, we propose an efficient small-object detection method, called FDA_YOLOv8, which is developed based on the YOLOv8s baseline and is designed to accurately detect small objects in UAV images under low computational cost. First, a four-head detection architecture is designed by introducing an additional lightweight detection head to enhance the sensitivity of smaller objects. Second, the dynamic head (Dyhead) framework is integrated to improve the representation capability of the detection head. Third, a FasterNet block is embedded into the C2f module to form the C2f_FA architecture, which improves spatial feature extraction while reducing model complexity and computational cost. Furthermore, an efficient multi-scale attention (EMA) mechanism is incorporated into the C2f_FA module, yielding the C2f_FE structure for better feature discrimination. Experiments on the VisDrone2019 dataset show that FDA_YOLOv8 achieves an mAP of 45.8%, outperforming YOLOv8s by 5.2%, confirming its effectiveness in refined small object detection for UAV imagery.

## Introduction

### Background

Small object detection (SOD) plays a critical role in security monitoring applications, especially in unmanned aerial vehicle (UAV) imagery. With the increasing availability of dataset and the rapid advancements in deep learning, object detection has achieved remarkable progress in recent years. SOD, which focuses on detecting small-sized objects, has emerged as a specialized field^1^. Typically, “small” objects are defined by an area threshold, such as objects with an area of 1024 pixels or fewer^2^. The widespread deployment of UAVs has significantly increased the demand for refined SOD, as UAV-based surveillance provides unique advantages such as high mobility, wide-area coverage, and real-time monitoring capabilities^3^. As a result, SOD for UAV imagery has become essential in various real-world applications, including traffic monitoring, security surveillance, disaster assessment, and environmental observation. However, due to the high-altitude perspective and limited image resolution, small objects in UAV imagery often appear blurred and indistinct, making them difficult to distinguish from complex backgrounds. Therefore, accurately localizing and classifying these objects remains a fundamental challenge, requiring more refined detection methods to enhance performance of UAV-based SOD.

Despite the advantages of UAV-based surveillance, several inherent limitations make accurate SOD highly challenging^4^. The high-altitude perspective reduces the apparent size of objects, making them harder to distinguish. Moreover, due to the elevated viewpoint and large scene coverage, small objects in UAV imagery often occupy only a limited number of pixels, which hampers the extraction of fine-grained features. Additionally, background clutter, poor contrast, and sensor noise often obscure small objects, leading to false positives and missed detections^5^. These challenges not only hinder the effectiveness of traditional object detection algorithms but also limit the performance of deep learning-based methods, which struggle to capture meaningful features for small-scale objects. Although multi-scale feature fusion and attention mechanisms have shown promise, their effectiveness often comes at the cost of increased model complexity and computation. Consequently, developing a refined detection method that effectively enhances feature representation while maintaining computational efficiency is essential for improving UAV-based SOD. Thus, achieving high-precision small object detection in UAV imagery remains a critical challenge, requiring the development of more efficient and robust detection frameworks that can effectively capture fine-grained features while maintaining low computational overhead to meet the resource constraints of UAV systems.

### Motivation

In the early stage of object detection, handcrafted feature-based methods are widely used^6^. For example, SVM-based human detection utilizing histogram of oriented gradient descriptors demonstrated superior performance over earlier feature sets. Because of the limited scale transformation ability, the method of handcrafted features performs weakness for small object detection. In recent years, deep learning-based object detection methods have demonstrated remarkable progress in handling multi-scale object detection, significantly outperforming traditional machine learning methods^7^. The method using feature pyramid network (FPN) enhance the feature representation of small object by fusion multi-scale features. Nevertheless, they rely heavily on upsampling and downsampling operations, and fail to fully retain fine-grained spatial details.

To address this, anchor-free detectors such as CornerNet have been used to eliminate the dependency on predefined anchor boxes, improving adaptability to varying object sizes. However, these methods struggle to localize small objects accurately, as keypoint-based representations tend to degrade under low-resolution conditions. Transformer-based object detection including Vision Transformers (ViTs) and DEtection TRansformers (DETR) has appeared, which adopt self-attention mechanisms to improve feature extraction across long-range dependencies. However, high computational costs make them less feasible for real-time UAV applications, where efficiency and lightweight deployment are critical concerns. The YOLO series has demonstrated fast inference speeds and competitive accuracy, making them suitable for UAV-based tasks. The YOLO-Drone model is an optimized YOLOv8 network for tiny UAV object detection^8^. In YOLO-Drone, a high-resolution detection head is introduced, while the large-object detection head and redundant network layers are removed. This modification enables faster and more effective detection of small objects^9^. However, these methods still face some limitations when applied to UAV imagery as follows.

1. Insufficient detection heads. Current models often use three or fewer detection heads, limiting their ability to accurately detect small objects.
2. Loss of fine-grained features. Upsampling operations in feature pyramids can cause feature degradation, making small object localization challenging.
3. High computational complexity. Many advanced feature extraction and attention mechanisms increase model complexity and inference time, which is not suitable for the UAV-based system with limited resources.

### Contributions

This study is dedicated to improving the precision of SOD for UAV imagery captured at high altitudes under real-world conditions. A refined framework called FDA_YOLOv8 is proposed to improve the detection accuracy while maintaining low computational cost. The main contributions of this study are summarized as follows.

1. A four-detection-head structure are presented to better capture fine-grained features of small objects. FDA_YOLOv8 introduces an additional detection head to the YOLOv8 structure. This allows the model to enhance feature extraction for small objects and improve detection accuracy in UAV imagery.
2. The dynamic head (Dyhead) is integrated into the detection head for enhancing feature representation. The Dyhead strengthen feature fusion across scales and locations, significantly boosts robustness against scale variations and cluttered environments.
3. To reduce computational cost while maintaining strong feature extraction capability, FDA_YOLOv8 integrates FasterNet (FA) blocks into traditional C2f blocks in YOLOv8, forming the C2f_FA architecture. Furthermore, the incorporation of an efficient multi-scale attention (EMA) mechanism within the FA-based module results in the C2f_FE architecture, further refining small object feature representation with minimal computational overhead.
4. The proposed FDA_YOLOv8 is evaluated on the VisDrone2019 dataset, a widely used benchmark for UAV-based object detection. Experimental results show that FDA_YOLOv8 achieves an mAP of 45.8%, surpassing YOLOv8s by 5.2%, demonstrating its superior capability in refined small object detection for UAV imagery. These results highlight the model’s effectiveness in balancing detection accuracy and computational efficiency, showing promising potential for real-time UAV deployment.

The rest of this paper is organized as follows. Section 2 summarizes related work on SOD for UAV imagery. Section 3 presents the methodology of the proposed FDA_YOLOv8 framework. Section 4 discusses the experimental setup and evaluates the results. Section 5 concludes the paper and outlines directions for future work.

## Related work

This section provides an overview of vision-based SOD methods, focusing on multi-scale feature extraction for small object detection and feature enhancement through dynamic structures and attention mechanisms.

### Multi-scale feature extraction for small object detection

In deep learning-based object detection, multi-scale feature extraction is essential to accurately detect objects of various sizes, especially small ones^10^. Two main detection frameworks–two-stage and one-stage–have been developed to enhance detection precision through multi-scale feature representations. Two-stage detection algorithms, such as R-CNN, Fast R-CNN^11^, and Faster R-CNN^12^, first generate region proposals and then perform classification and localization on each candidate region. These methods achieve high accuracy and perform well in complex scenes with multiple objects. By leveraging multi-scale region proposals and deep feature extraction, two-stage algorithms can capture small object details more effectively. However, their high computational cost and poor real-time performance significantly limit their applicability in UAV-based systems, where efficient processing and limited onboard resources are critical constraints.

To meet real-time processing requirements, one-stage detection algorithms, such as You Only Look Once (YOLO) and Single Shot Detector (SSD), are widely used in real-time object detection tasks due to their fast inference speed. They bypass the region proposal generation by predicting object classes and bounding boxes in a single forward pass through the network^13^. The YOLO series has made continuous improvements in speed, accuracy, and efficiency. For example, YOLOv3 introduced multi-scale detection by adding multiple detection heads, and YOLOv5 improved architecture design and anchor mechanisms to reduce model size and enhance inference efficiency. However, these models still struggle with small object detection due to their reliance on fixed-size anchors and coarse feature maps, which lack the resolution necessary to distinguish small-scale features. To improve multi-scale representation, various feature fusion strategies such as FPN, Path Aggregation Network (PANet), and BiFPN have been proposed. These methods aggregate features from different network layers to preserve both semantic context and fine-grained spatial details. While effective, they may still suffer from feature degradation due to repeated upsampling. Although YOLOv8 enhances feature representation and simplifies model configuration, its perrformance in detecting small objects for UAV imagery remains limited due to low-resolution inputs and insufficient granularity in feature extraction.

More recently, Transformer-based architectures such as DETR have demonstrated strong potential by modeling long-range dependencies through self-attention mechanisms^14^. While these models show competitive performance on large-scale benchmarks, their high computational cost and memory footprint pose significant challenges for real-time UAV deployment, where latency and power consumption are tightly constrained. Complementary strategies such as attention-based context modeling, enhanced receptive field design, and background suppression modules have also been proposed to improve the distinguishability of small targets. However, many of these methods either increase the model complexity or rely on high-resolution input, which conflicts with the resource limitations in UAV platforms.

In UAV imagery specifically, small object detection is further complicated by motion blur, low contrast, and complex backgrounds. Most current one-stage detectors employ three detection heads, which may not provide sufficient granularity to resolve fine details of small targets. Thus, designing refined architectures with enhanced multi-scale representation and low computational overhead remains an active and essential direction for improving small object detection in UAV-based systems.

### Feature enhancement using dynamic structures and attention mechanisms

Accurate SOD in UAV imagery hinges on the model’s ability to extract and represent fine-grained features from complex scenes. To this end, dynamic feature enhancement frameworks have gained significant attention^15^. These frameworks adaptively modulate the feature extraction and fusion process based on the semantic complexity of input data. For instance, the DyHead architecture^16^ dynamically fuses multi-level and multi-scale features across layers and detection heads, enabling the network to attend to both local details and global context. This flexibility enhances robustness to variations in object scale, shape, and background clutter, which are prevalent in UAV surveillance imagery. However, the increased structural complexity may introduce latency and memory overhead, limiting deployment on UAV platforms that require real-time processing and low-power consumption.

In addition to dynamic structures, attention mechanisms have become a crucial component for enhancing small object detection. Early methods, such as Squeeze-and-Excitation blocks, enhance channel-wise attention by reweighting features adaptively^17^. More advanced strategies include multi-head self-attention and Transformer-based backbones, such as ViT and DETR, which model long-range dependencies and global context more effectively. These methods have shown promise in dense and cluttered scenes; however, their high computational demands hinder their suitability for UAV deployment, where latency, memory, and power consumption are constrained. To address this, lightweight attention modules have been introduced. For example, EMA enables effective multi-scale feature aggregation with minimal computational overhead, making it suitable for edge devices^18^. EMA emphasizes discriminative features across spatial and scale dimensions while preserving efficiency, thus outperforming in both detection accuracy and inference speed. When integrated with dynamic head structures, such attention modules can significantly enhance the model’s sensitivity to small-scale visual cues. Therefore, combining dynamic feature fusion with lightweight attention mechanisms constitutes a promising direction for improving small object detection in UAV-based real-time systems.

## Proposed method

This section first provides an overview of the proposed method and then highlights its key innovations, including efficient feature extraction with C2f_FA architecture, refined small object representation with C2f_FE architecture, dynamic integration for enhancecd feature fusion, and four-detection-head structure for fine-grained object detection.

### Overview of FDA_YOLOv8

**Fig. 1:**
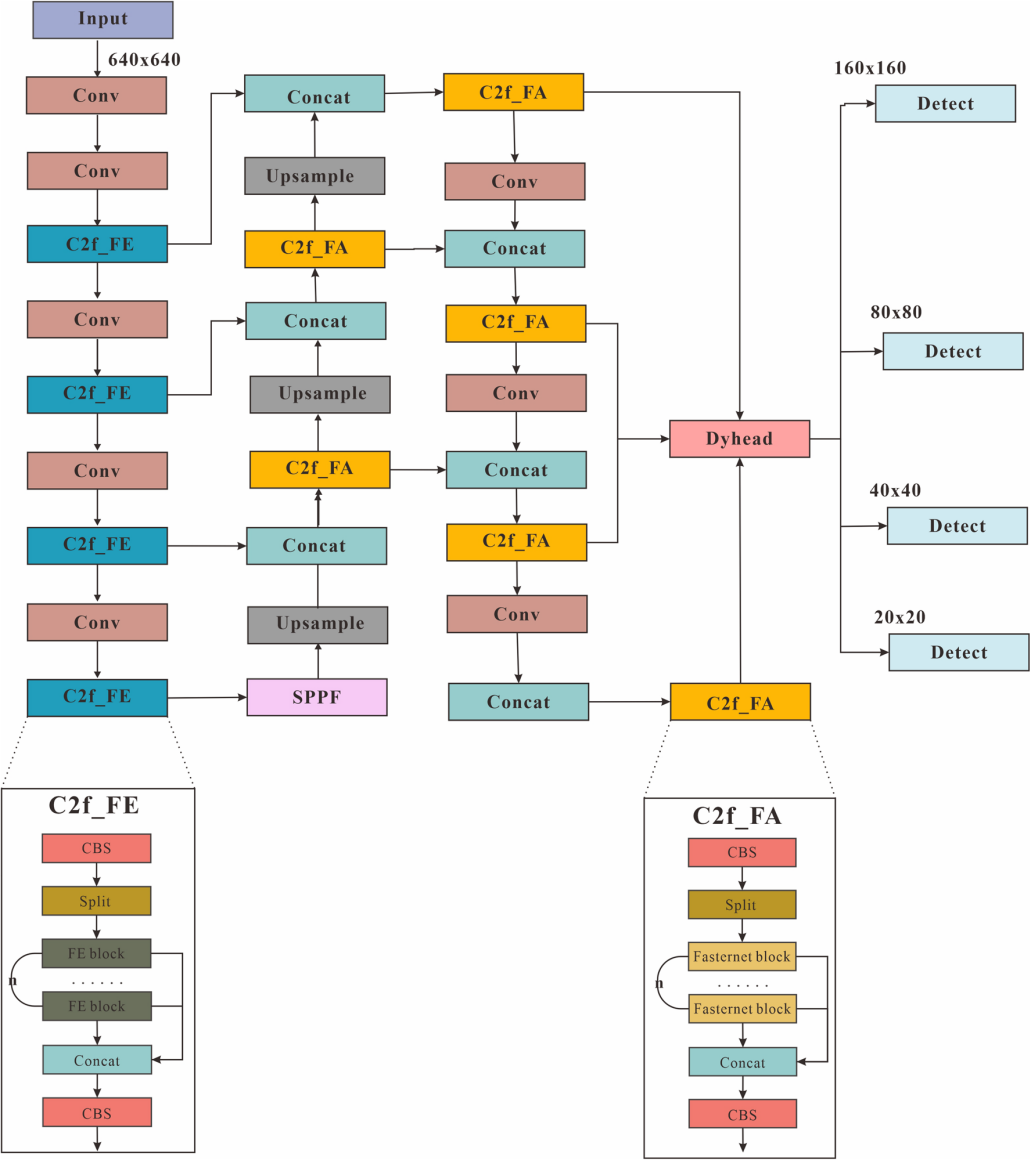
Structure of the FDA_YOLOv8 model.

Figure 1 illustrates the overall architecture of the proposed FDA_YOLOv8. The model is built on the YOLOv8s baseline, and all architectural improvements in this study are implemented on this version. FDA_YOLOv8 adopts a feature pyramid architecture designed for object detection. Given a 640*×*640 input image, the model first extracts multi-scale features through a hierarchical convolutional backbone. The backbone consists of multiple C2f_FE modules, which progressively refine feature representations, followed by an SPPF (Spatial Pyramid Pooling Fast) module to enhance multi-scale information encoding. The extracted features are then fused by several C2f_FA modules through upsampling and concatenation operations, enabling effective multi-scale feature integration across different resolutions. This design ensures effective feature fusion and preserves critical object-related information across multiple resolutions.

To further refine the detection process, the model incorporates a Dyhead module, which dynamically enhances feature selection and improves robustness across different scales. The detection head outputs predictions at four different resolutions (160*×*160, 80*×*80, 40*×*40, and 20*×*20), allowing for accurate detection of objects at varying sizes. This architecture combines efficient feature extraction, hierarchical feature aggregation, and dynamic adaptation in the detection head, demonstrating a favorable balance between detection accuracy and inference speed for UAV image detection scenarios.

### Efficient feature extraction with C2f_FA Architecture

The C2f_FA module in FDA_YOLOv8 is designed to optimize feature extraction while reducing computational overhead. Unlike C2f_FE, which enhances feature extraction with a focus on fine-grained small object representation, C2f_FA prioritizes efficient multi-scale feature aggregation by integrating FasterNet blocks. By utilizing a lightweight convolutional design with PConv and PWConv layers, C2f_FA significantly improves computational efficiency while maintaining high detection accuracy.

As shown in Fig. 1, C2f_FA follows a structured processing pipeline. It begins with an initial CBS block, followed by a *Split* operation, which partitions the input feature maps into multiple branches. These branches are processed through stacked FasterNet Blocks, which incorporate Partial Convolution (PConv) and Pointwise Convolution (PWConv, 1*×*1 Conv) to optimize computational efficiency. The *PConv* layer selectively applies standard convolution to only a portion of the input channels, drastically reducing FLOPs and memory access costs while preserving spatial feature integrity^19^. Compared to conventional convolutional layers, PConv achieves a 16-fold reduction in computational complexity, making it highly suitable for real-time object detection tasks. After processing through *n* FasterNet blocks, the aggregated features are concatenated and further refined through a final *CBS* operation to ensure smooth feature integration.

By leveraging FasterNet-based feature aggregation, C2f_FA improves the computational efficiency of FDA_YOLOv8 while preserving high-quality feature representations. This structure is particularly beneficial for large-scale detection scenarios, where computational resources are a key constraint. The lightweight yet effective design of C2f_FA enables the model to achieve a superior trade-off between detection accuracy and real-time processing capability, solidifying its role as a crucial component in the FDA_YOLOv8 framework.

### Refined small object representation with C2f_FE architecture

**Fig. 2:**
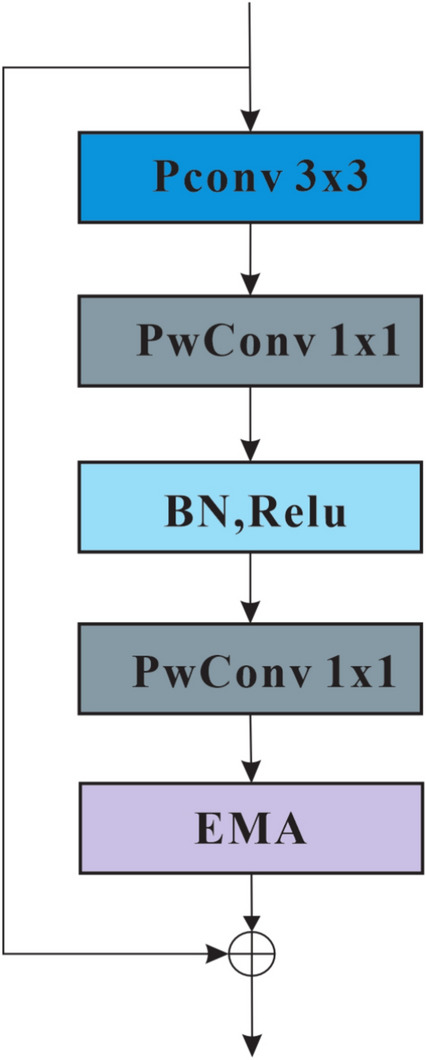
Architecture of FNE.

The C2f_FE module in FDA_YOLOv8 is designed to enhance feature extraction efficiency, particularly for small object detection. Channel and spatial attention mechanisms have proven highly effective in enhancing feature discriminability across various computer vision tasks. However, reducing channel dimensionality to model cross-channel relationships may negatively impact the extraction of deep visual representations. To addres this, this study incorporates an efficient multi-scale attention (EMA) module after the FasterNet Block, forming the FNE block to refine feature representations efficiently. In this process, input features first undergo partial convolution within the FNB block to minimize unnecessary computations caused by channel redundancy. Subsequently, channel dimensions are grouped and subdivided into multiple sub-features, ensuring a uniform distribution of functions within each functional group in spatial semantics. The structures of FNE is shown in Fig.  2.

**Fig. 3:**
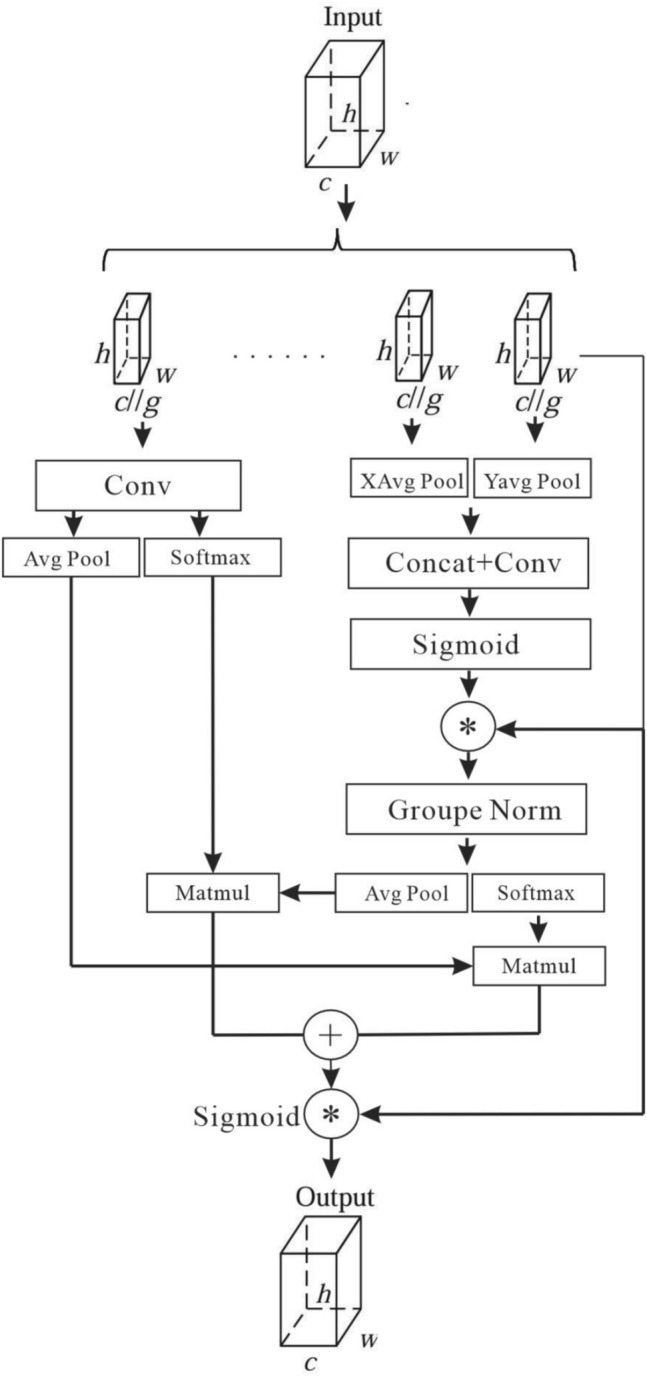
Architecture of EMA.

As illustrated in Fig.  1, C2f_FE in begins with an initial *CBS* (Convolution-BatchNorm-SiLU) block, followed by a *Split* operation, which divides the feature maps into multiple parallel branches. Each branch is processed through stacked FNE blocks, which incorporate EMA to enhance spatial and channel feature representations. The EMA module, as shown in Fig.  3, consists of feature grouping, parallel sub-networks, and cross-spatial learning, ensuring efficient feature refinement^18^. The FNE block utilizes FastNet-based feature extraction with grouped convolution, depthwise separable convolution, and channel pruning, effectively capturing local and global spatial semantics. After processing through *n* stacked FNE blocks, the extracted features are concatenated and refined via a final *CBS* operation.

By integrating EMA within C2f_FE, FDA_YOLOv8 significantly enhances small object feature representation. This ensures that fine-grained details are effectively preserved and leveraged for detection, particularly in UAV-based imagery, where small object recognition is a critical challenge. The hierarchical structure of C2f_FE not only improves detection accuracy but also maintains computational efficiency, making it a vital component of the FDA_YOLOv8 architecture.

### Dynamic integration for enhancecd feature fusion

**Fig. 4:**
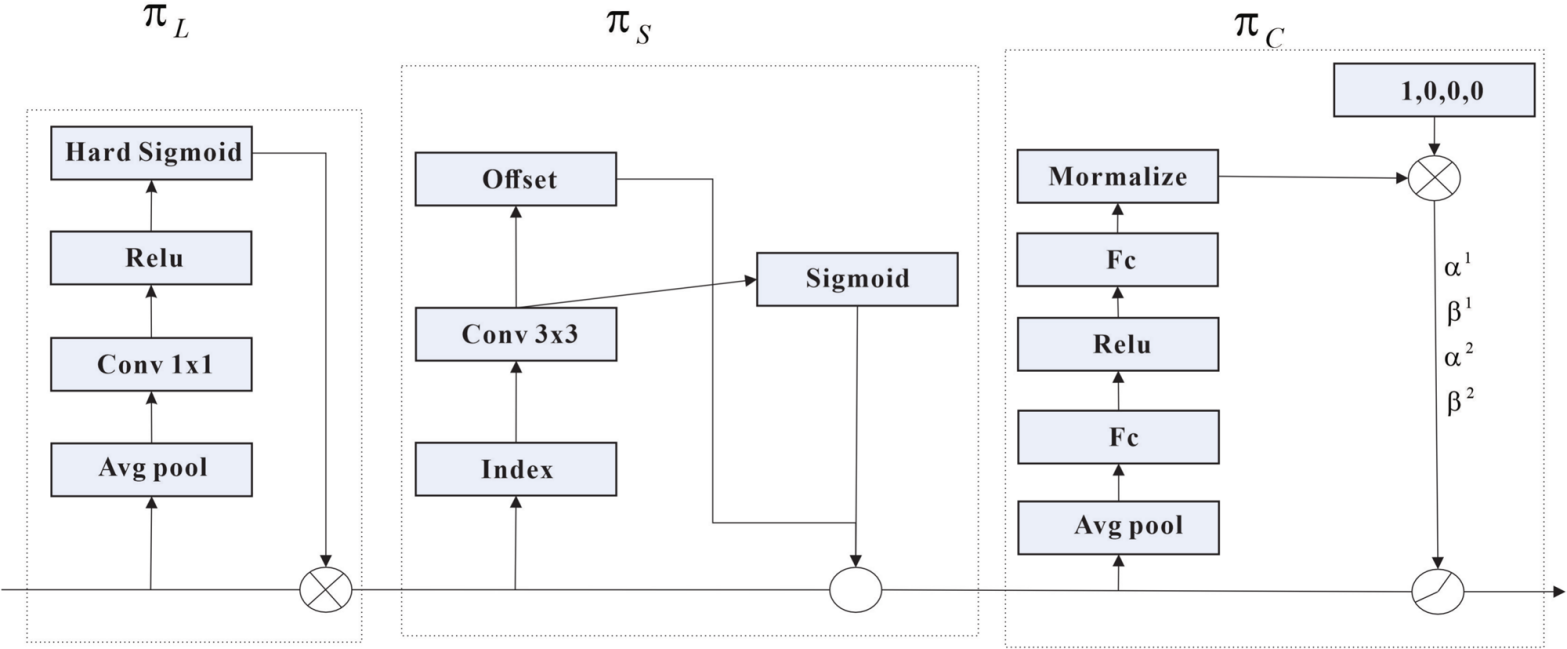
Architecture of DyHead.

The DyHead module in FDA_YOLOv8 plays a crucial role in improving feature fusion by dynamically integrating scale-aware, spatial-aware, and task-aware attentions. This module enhances object detection by incorporating multi-level feature fusion while maintaining high adaptability to objects of varying scales, spatial locations, and detection tasks. By unifying three types of attention mechanisms into a single framework, DyHead enhances the robustness and representation capacity of the detection head^14^. The DyHead structure, as shown in Fig.  4, consists of three key components: scale-aware attention ($\pi _L$), spatial-aware attention ($\pi _S$), and task-aware attention ($\pi _C$), each of which operates sequentially to refine feature representations. In our framework, DyHead is integrated after the C2f_FA modules (as shown in Fig.  1) to further optimize the refined multi-scale features before final detection. This dynamic feature modulation enables superior detection performance, particularly for small objects in complex environments.

Before features are fed into the DyHead detection head, the feature pyramid network first scales the backbone outputs, ensuring consistent feature scales across different levels. The backbone’s multi-scale output is structured as a four-dimensional tensor $O_b \in R^{L \times H \times W \times C}$, where *L* represents the number of pyramid levels, and *H*, *W*, *C* denote the height, width, and channel dimensions of the features. To facilitate DyHead’s processing, this tensor is reshaped into a three-dimensional form ${O_b} \in R^{L \times S \times C}$, where *S = H × W*, allowing DyHead to efficiently process scale, spatial, and channel dimensions. Each attention module in DyHead ($\pi _L$, $\pi _S$, and $\pi _C$) operates on a distinct dimension, refining feature representations in a sequential manner.

1 $$\begin{aligned} W(O_b)=\pi _C\left( \pi _S\left( \pi _L({O_b}) \cdot {O_b}\right) \right) . \end{aligned}$$

This cascaded processing structure enables DyHead to capture multi-scale, spatial, and task-related contextual information effectively, ultimately boosting object detection accuracy.

### Four-detection-head structure for fine-grained object detection

The four-detection-head structure in FDA_YOLOv8 is specifically engineered to improve the detection accuracy of small and fine-grained objects. Unlike traditional models that use a single detection head, this architecture utilizes multiple heads operating at different scales to improve object detection across various sizes. Each detection head processes features at distinct resolutions, with larger heads focusing on detecting large objects and smaller heads optimized for fine-grained, small object detection. This multi-scale design ensures that the model captures both large and small object features, addressing the challenge of detecting small objects in complex and cluttered environments, such as those found in UAV imagery.

The four-detection-head structure improves detection precision, especially for small objects that are typically underrepresented in lower-resolution feature maps. By focusing on different levels of feature abstraction, the model refines small object representations, ensuring better accuracy. This innovative design leads to a robust and efficient system that balances detection accuracy and computational efficiency, making it highly effective for real-time object detection tasks.

## Experiments

This section introduces the dataset, evaluation metrics, and experimental setup, including model configurations and training details. It also presents ablation studies to evaluate the contributions of individual components, as well as comparative experiments with existing methods. In all experiments, YOLOv8s is used as the baseline model for architectural modifications and performance comparisons unless otherwise specified.

### Dataset

The VisDrone2019 dataset is a widely used benchmark for UAV-based object detection and serves as the basis for evaluating the performance of the proposed FDA_YOLOv8 method in this study^20^. Although the images in VisDrone2019 have high absolute resolution and are captured from drones flying over urban environments, many targets still occupy only a few pixels because of the aerial viewpoint and broad scene coverage. Therefore, limited effective object-level resolution remains a major challenge for small object detection in this dataset. In addition, the dataset contains objects at varying scales, with a significant proportion being small, making it a representative benchmark for evaluating small object detection algorithms in real-world aerial imagery.

The VisDrone2019 dataset includes 10,209 static images captured at different UAV heights, with 6,471 training images, 548 validation images, and 3,190 test images, containing a total 2.6 million object instances. The images were captured under diverse conditions, including varying weather scenarios, times of day, and camera motion levels, further enhancing the dataset’s realism and complexity. Each image is annotated with bounding boxes marking the locations and sizes of objects, with labels corresponding to various categories, such as pedestrians (standing or walking persons), people (persons in other postures), cars, vans, buses, trucks, motorcycles, bicycles, awning tricycles, and tricycles. These detailed annotations facilitate thorough evaluation of object detection models, particularly in challenging scenarios involving occlusion, scale variation, and complex environments.

### Evaluation metrics

The performance of the proposed model is evaluated using several key metrics, including precision (P), recall (R), mean Average Precision (mAP), model parameters (Param), and Floating Point Operations (FLOPs). Among them, mAP is adopted as the primary metric for evaluating detection accuracy. In this study, we mainly report mAP@0.5, where a predicted bounding box is regarded as a true positive when its Intersection-over-Union (IoU) with the corresponding ground-truth box is greater than or equal to 0.5. In addition, Param and FLOPs are used to evaluate the model size and computational complexity, respectively. Average Precision (AP) is computed as the area under the precision-recall curve for each class, while mAP is obtained by averaging the AP values over all classes. Specifically, the AP of the *i*-th class is defined as

2 $$\begin{aligned} AP_i = \int _0^1 P_i(R)dR, \end{aligned}$$

where $P_i(R)$ denotes the precision of the *i*-th class at recall *R*. Therefore, the mAP is computed as

3 $$\begin{aligned} mAP = \frac{1}{N}\sum _{i=1}^{N} AP_i, \end{aligned}$$

where *N* denotes the number of object categories.

Precision measures the proportion of correctly detected objects among all predicted objects, while recall measures the proportion of correctly detected objects among all ground-truth objects. They are defined as

4 $$\begin{aligned} P = \frac{TP}{TP + FP}, \end{aligned}$$

5 $$\begin{aligned} R = \frac{TP}{TP + FN}, \end{aligned}$$

where *TP*, *FP*, and *FN* represent true positives, false positives, and false negatives, respectively.

The number of parameters indicates the model size and storage requirements. FLOPs quantify the number of floating-point operations required for a single forward inference. A lower FLOPs value generally indicates lower computational complexity, which is beneficial for real-time and resource-constrained applications.

### Experimental settings

The experimental environment is the Ubuntu 16.04 operating system, and the network of NVIDIA RTX 3090 GPU with 24 GB of video memory is used for the experimental operation. And using Python 3.8, and using Pytorch version 2.0.1 as experimental deep learning frameworks,and take advantage of coco’s pre-trained weights because of the similarity in the tasks of using the YOLO model for SOD and object detection on the coco dataset.

Properly configuring network parameters can markedly elevate the effectiveness of algorithmic training and detection. In this study, the size of input images is *640 × 640*, with a batch size of 64, an initial learning rate of 0.01. We iterate through a total of 200 epochs, with a specified Intersection over Union (IoU) threshold of 0.5 for Non-Maximum Suppression (NMS).

### Ablation study

In this section, we present an ablation study to evaluate the contributions of key components in the FDA_YOLOv8 model. Specifically, we analyze the impact of the four-detection-head structure, the DyHead integration, and the FasterNet-based C2f modules on the model’s performance. The experiment results are summarized in Table 1, which compares different configurations based on several performance metrics.

#### Effect of four-detection-head structure on model performance

In the first ablation study, we assess the impact of adding an extra detection head in the FDA_YOLOv8 model. The standard YOLOv8 model, shown in the first row of Table 1, uses three detection heads. By introducing a four-detection-head structure (as shown in the second row of Table 1), the model significantly enhances its ability to capture features across multiple scales. The introduction of the additional detection head improves performance, increasing the mAP from 40.7% to 44.2%. This enhancement is especially effective for detecting small objects, which is a key challenge in UAV imagery where object sizes and distances can vary significantly.

#### Effect of DyHead integration on model performance

Building on the previous ablation study with the four-detection-head structure, we evaluate the effect of integrating the DyHead module. As shown in the third row of Table 1, DyHead increases the mAP from 44.2% to 45.6%, demonstrating its contribution to enhanced detection performance.The improvement comes from DyHead’s dynamic feature modulation, which enhances feature fusion across scales and locations. This allows the model to better handle scale variations and clutter, key challenges in UAV imagery. By applying dynamic attention at multiple levels, DyHead enables the model to focus on relevant features, improving detection, especially for small objects affected by size, occlusion, or distance. Overall, DyHead boosts the model’s robustness, leading to more accurate detection in complex environments.

#### Effect of C2f_FA architecture on model performance

In this ablation study, we evaluate the effect of replacing the traditional C2f blocks in YOLOv8 with FA blocks, resulting in the C2f_FA architecture. The primary goal of this modification is to reduce computational cost while maintaining high feature extraction performance. As shown in the fourth row of Table 1, replacing C2f blocks with FasterNet results in a significant reduction in computational complexity, with FLOPs decreasing from 42.9G to 38.7G. Despite this reduction, the mAP remains stable at 45.8%, indicating that the C2f_FA architecture achieves a favorable balance between accuracy and computational efficiency.

#### Effect of C2f_FE architecture on model performance

This experiment evaluates the optimization introduced by integrating the multi-scale attention mechanism into the FA-based module, resulting in the C2f_FE architecture. As shown in the fifth row of Table 1, the addition of EMA enhances the model’s ability to refine small object feature representations while keeping computational overhead minimal. This results in a slight increase in mAP, reaching 45.8%. Compared to the previous configuration (row 4), the C2f_FE architecture achieves similar mAP but with improved computational efficiency. Specifically, it reduces FLOPs from 42.9G to 34.8G and lowers model parameters from 10.70M to 7.87M, maintaining strong performance while optimizing resource usage.

**Table 1:** Performance comparison of FDA_YOLOv8 with various configurations.

Module configurations					Performance metrics		
*Yolov*8*s*	*Four* *head*	*Dyhead*	*FA*	*FNE*	*Flops* (*G*)	*Param* (*M*)	*mAP~(%)*
*\checkmark*					28.6	11.20	40.7
*\checkmark*	*\checkmark*				37.0	10.60	44.2
*\checkmark*	*\checkmark*	*\checkmark*			42.9	10.70	45.6
*\checkmark*	*\checkmark*	*\checkmark*	*\checkmark*		38.7	9.28	45.8
*\checkmark*	*\checkmark*	*\checkmark*	*\checkmark*	*\checkmark*	34.8	7.87	45.8

Based on the analysis mentioned above, integrating the DyHead module with the four-detection-head structure in the YOLOv8s model improves performance, increasing mAP from 44.2% to 45.6%. This change enhances feature fusion and improves the handling of scale variations and cluttered environments. The C2f_FA and C2f_FE configurations not only achieve a slightly higher mAP of 45.8%, but also significantly reduce FLOPs and model parameters, offering a favorable balance between computational efficiency and accuracy. These results demonstrate the effectiveness of the optimized architecture of FDA_YOLOv8 in enhancing both performance and efficiency.

#### Convergence analysis

**Fig. 5:**
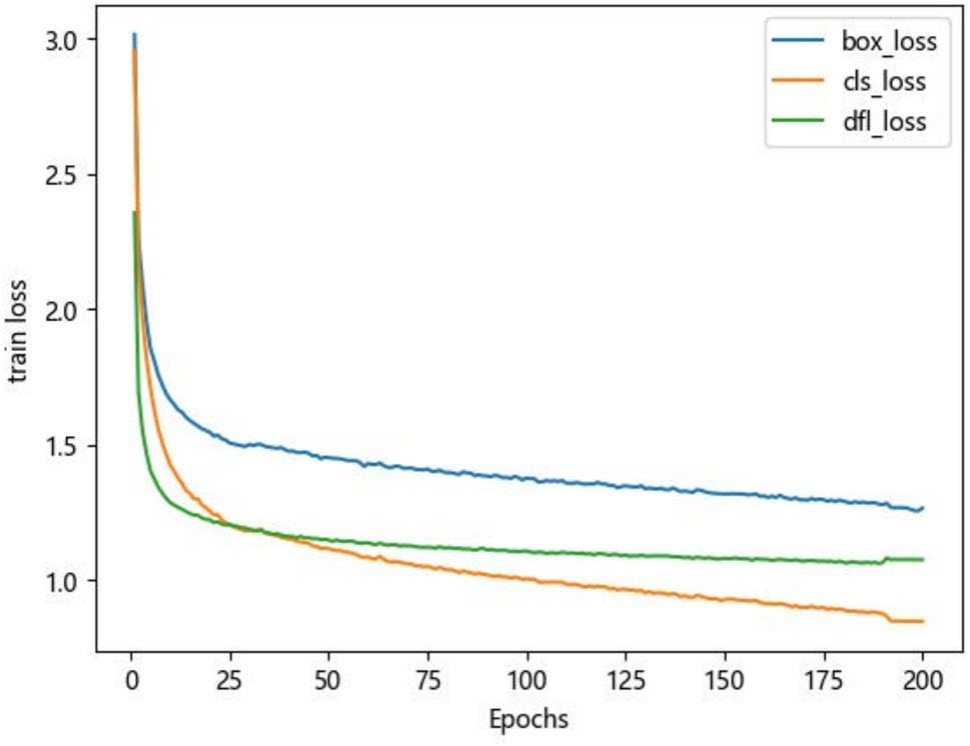
Loss with varying epochs during model training. .

This experiment analyzes the convergence of FDA_YOLOv8 based on the ablation study results. Figure 5 shows the training loss curves for three components: box_loss, cls_loss, and dfl_loss across the number of epochs. From the figure, it is evident that all three losses rapidly decrease in the first 25 epochs. This suggests that the FDA_YOLOv8 model quickly adapts to the UAV-based detection task, learning the relevant features efficiently in the early stages of training. After the initial drop, the losses start to stabilize and decrease more gradually. This indicates that the model has reached a point where learning becomes more incremental and fine-tuned. Around the $150^{th}$ epoch, all loss curves level off, suggesting that the model has converged to a solution with minimal further improvements. This indicates that the model has effectively learned the relevant features. The FDA_YOLOv8 model exhibits fast convergence during the early epochs, followed by stable convergence of all loss components. These findings highlight the efficiency of the FDA_YOLOv8 architecture in both feature extraction and model optimization, confirming the effectiveness of the proposed method for small object detection in UAV imagery.

### Comparison with existing methods

To assess the performance of the proposed FDA_YOLOv8, we compare it with existing methods, including SSD^13^, YOLOv5s^21^, YOLOv7-Tiny^22^, YOLOv8s^23^, YOLOv12s^24^, YOLOv26s^25^, Faster R-CNN^12^, and YOLO-UAV^9^. The comparison results are summarized in Table 2.

The results reveal a clear trade-off between detection accuracy and model complexity across different methods. SSD, as a classical one-stage detector, achieves only 15.2% mAP, demonstrating limited effectiveness for UAV small-object detection. Similarly, Faster R-CNN achieves 37.2% mAP, but requires 14.2M parameters, which is still considerably larger than the proposed FDA_YOLOv8. Among lightweight YOLO-based models, YOLOv5s achieves an mAP of 34.5%, and YOLOv7-Tiny reaches 36.4%, both showing moderate performance. YOLOv8s, a more advanced variant, achieves 40.6% mAP, benefiting from improved feature representation. We also compare with two recent YOLO versions, YOLOv12s and YOLOv26s. YOLOv12s yields 37.1% mAP with 9.3M parameters, while YOLOv26s achieves 37.2% mAP with 9.5M parameters. Compared with these recent YOLO models, FDA_YOLOv8 achieves clearly higher detection accuracy while using fewer parameters.

The proposed FDA_YOLOv8 achieves 45.8% mAP with only 7.8M parameters, striking a favorable balance between detection performance and model compactness. Compared to YOLOv8s, FDA_YOLOv8 improves mAP by 5.2% while using 30.4% fewer parameters, demonstrating the effectiveness of the proposed enhancements, such as multi-scale feature aggregation and attention mechanisms. Although YOLO-UAV achieves the highest mAP with 46.7%, it requires 9.8M parameters, which is 25.6% larger than ours, and its FLOPs (47.5) are also considerably higher than those of FDA_YOLOv8 (34.8). Therefore, the proposed model offers a more favorable trade-off between detection accuracy and computational cost, making it a practical solution for efficient UAV-based object detection.

These results demonstrate that FDA_YOLOv8 effectively enhances UAV small object detection and consistently outperforms recent YOLO variants in mAP while using fewer parameters. Its ability to maintain competitive detection performance with a compact model size makes it well suited for resource-constrained UAV deployment scenarios.

**Table 2:** Performance comparison of FDA_YOLOv8 with existing methods.

Method	*mAP@0.5~(%)*	Param (*M*)	*P~(%)*	*R~(%)*	FLOPs
SSD	15.2	24.6	28.4	19.8	31.4
Yolov5s	34.5	7.2	46.3	32.1	16.5
Yolov8s	40.6	11.2	52.1	39.5	28.6
YOLOv7-Tiny	36.4	6.2	48.7	32.4	35.4
Faster R-CNN	37.2	14.2	48.9	34.7	23.8
YOLO-UAV	46.7	9.8	55.7	40.8	47.5
YOLOv12s	37.1	9.3	47.8	37.8	21.4
YOLOv26s	37.2	9.5	52.5	38.0	20.7
FDA_YOLO (Ours)	45.8	7.8	56.0	43.5	34.8

### Real-time performance analysis

To further evaluate the real-time performance of the proposed method, FPS was measured on an NVIDIA RTX 3090 GPU with a batch size of 1 and an input resolution of *640 × 640*. The reported FPS includes model inference and post-processing, while excluding image loading and visualization time. As shown in Table 3, FDA_YOLOv8 achieves the highest FPS of 60.3 while also obtaining the best mAP@0.5 of 45.8%. Compared with YOLOv8s, the proposed method improves the detection accuracy from 40.6% to 45.8% and increases the inference speed from 55.1 FPS to 60.3 FPS, indicating that the proposed method achieves a better balance between detection accuracy and real-time inference performance.

**Fig. 6:**
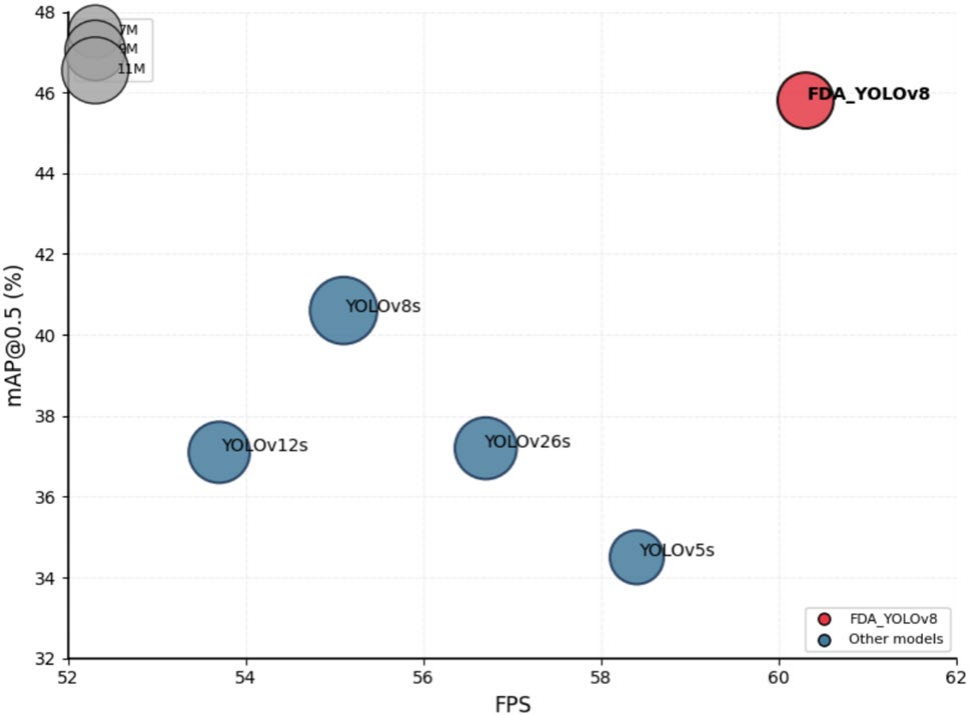
Accuracy–efficiency–model size trade-off on the VisDrone2019 dataset.

In addition, a bubble chart is provided to intuitively illustrate the trade-off among detection accuracy, inference speed, and model size. As shown in Fig.  6, the x-axis denotes FPS, the y-axis denotes mAP@0.5, and the bubble size indicates the number of model parameters. FDA_YOLOv8 is located in the upper-right region with a relatively small model size, demonstrating its superior accuracy–efficiency–model size trade-off.

**Table 3:** Comparison of accuracy, model size, and inference speed on the VisDrone2019 dataset.

Method	*mAP@0.5~(%)*	Param (*M*)	FPS
YOLOv5s	34.5	7.2	58.4
YOLOv8s	40.6	11.2	55.1
YOLOv12s	37.1	9.3	53.7
YOLOv26s	37.1	9.5	56.7
FDA_YOLOv8	45.8	7.8	60.3

### Model performance analysis

To further analyze the performance of FDA_YOLOv8, we compare its category-wise detection accuracy against YOLOv8 and evaluate its classification consistency using a confusion matrix.

**Fig. 7:**
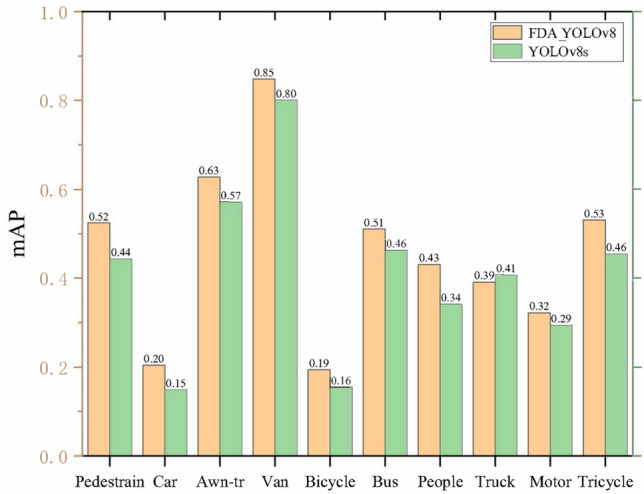
Classification results using FDA_YOLOv8 and YOLOv8s on the VisDrone2019 dataset..

**Category-wise performance analysis.** Figure 7 illustrates the detection accuracy of FDA_YOLOv8 and YOLOv8s across different object categories. It is evident that FDA_YOLOv8 outperforms YOLOv8s in most categories, particularly in detecting pedestrians, awning tricycles, vans, and tricycles, which are often challenging due to their small size and occlusions in UAV imagery. This improvement can be attributed to the enhanced feature extraction and multi-scale attention mechanisms introduced in FDA_YOLOv8. Although the gains vary across categories, the general trend shows a consistent performance boost, confirming the model’s superiority in small and occluded object detection.

**Fig. 8:**
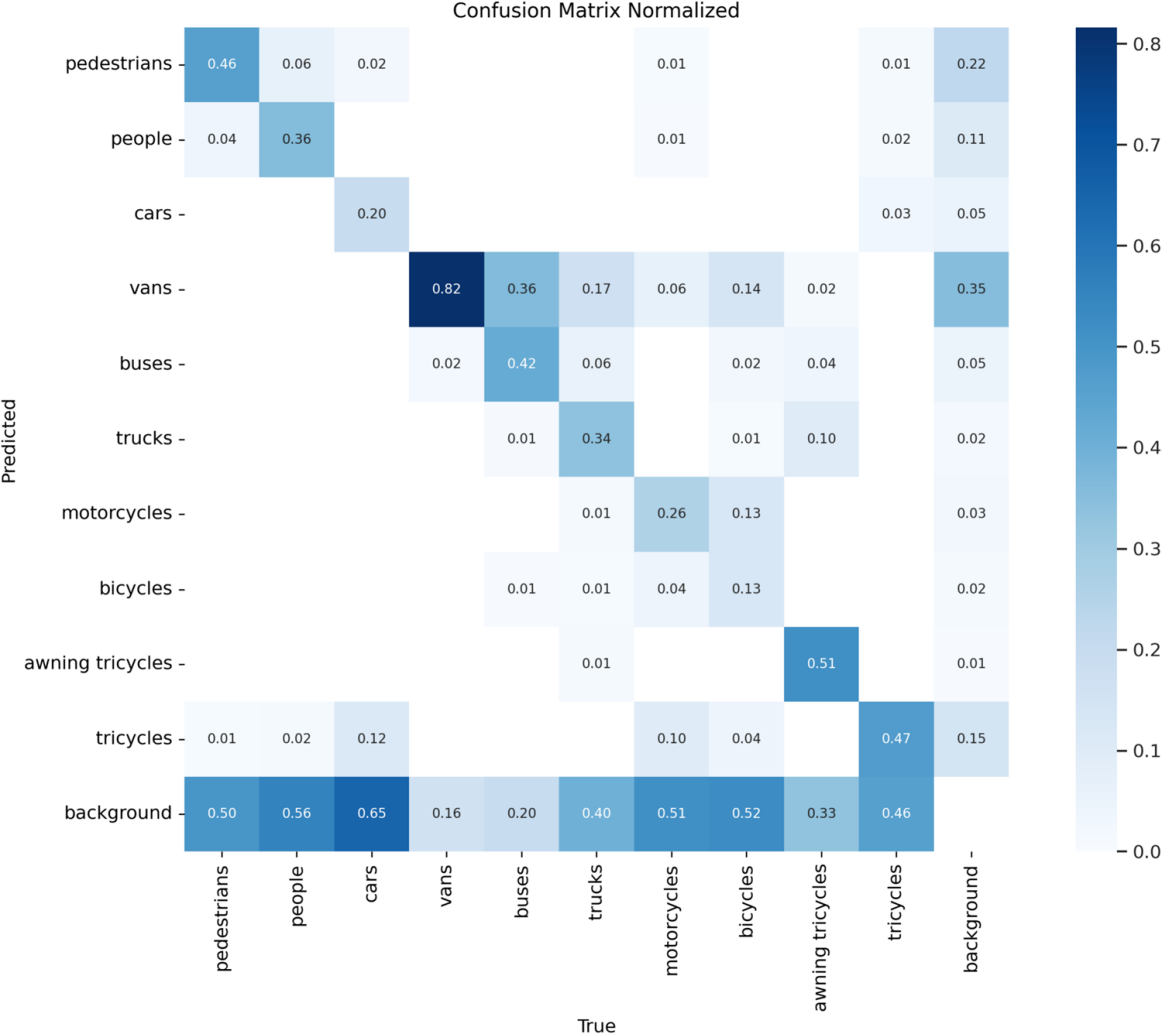
Confusion matrix of FDA_YOLOv8 on the VisDrone2019 dataset..

**Confusion Matrix Analysis.** Figure 8 provides a confusion matrix to further analyze the classification reliability of FDA_YOLOv8. The diagonal values represent correct classifications, while off-diagonal values indicate misclassifications. We observe that the model performs well in distinguishing larger, well-separated categories such as vans with 82% mAP and awning tricycles with 51%, demonstrating strong feature representation. However, misclassification rates increase for visually similar objects, such as pedestrians misclassified as people and trucks confused with buses. These errors highlight challenges in differentiating objects with similar visual characteristics, which may be mitigated through additional fine-tuning or higher-resolution input data.

The FDA_YOLOv8 model demonstrates generally improved category-wise detection accuracy compared to YOLOv8s. The confusion matrix shows strong classification for well-separated objects (e.g., vans, awning tricycles), while revealing expected confusion between visually similar pairs (e.g., pedestrian vs. people, truck vs. bus). These results validate the effectiveness of FDA_YOLOv8 in enhancing detection accuracy and classification reliability in complex UAV-based scenarios.

### Visualization of FDA_YOLOv8-based detection

**Fig. 9:**
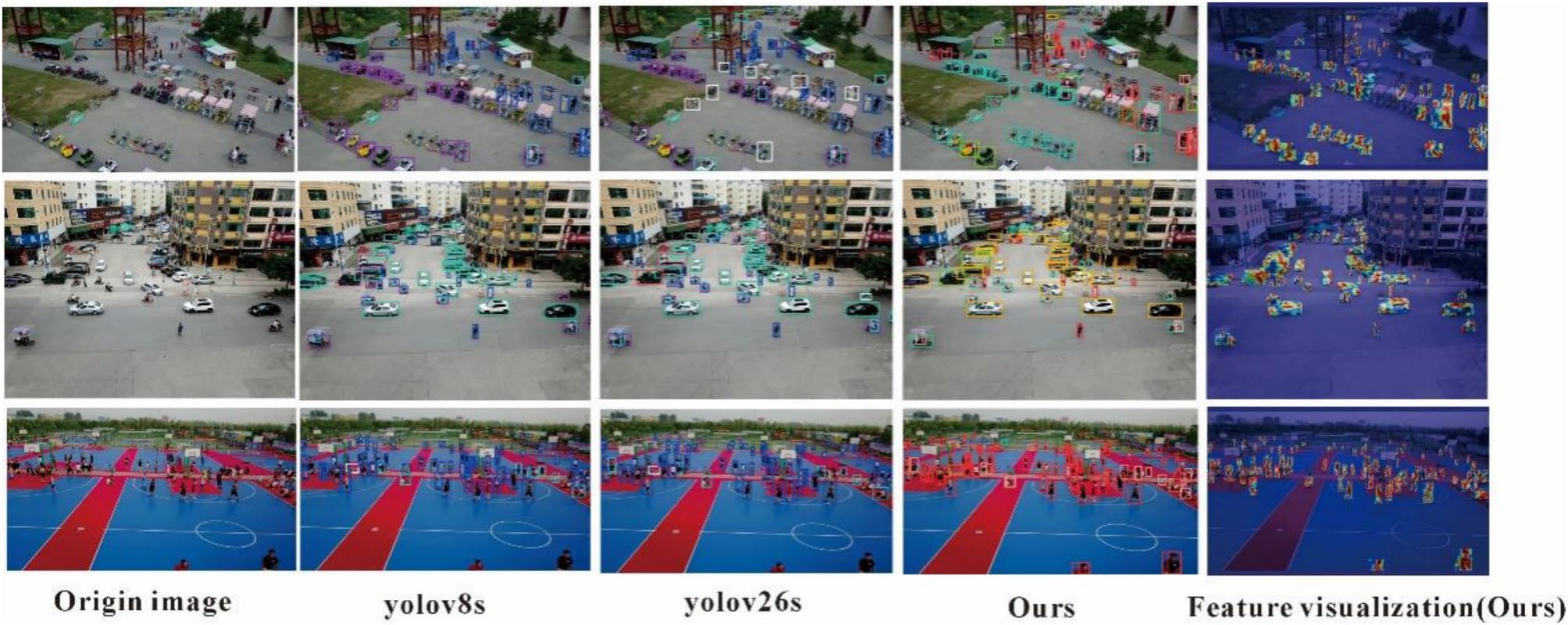
Qualitative comparison of detection results produced by YOLOv8s, YOLOv26s, and the proposed FDA_YOLOv8 on representative UAV-perspective images, together with the Grad-CAM-based feature visualization of FDA_YOLOv8.

To further investigate the effectiveness of FDA_YOLOv8, we provide both qualitative comparisons of detection results and feature visualization analysis on representative UAV-perspective images. As shown in Fig.  9, the visual comparison includes the original images, the detection results of YOLOv8s, YOLOv26s, and the proposed FDA_YOLOv8, together with the Grad-CAM-based feature visualization of FDA_YOLOv8. Grad-CAM is employed to generate the heatmaps by backpropagating the gradients of the target output, which helps reveal the spatial regions that contribute most to the model prediction^26^. In the generated heatmaps, regions with stronger responses are highlighted in red, while less activated regions are shown in blue.

From the visual comparison results, it can be observed that FDA_YOLOv8 detects more small and densely distributed objects in complex UAV scenes, while showing fewer missed detection than YOLOv8s and YOLOv26s. In particular, for distant targets and crowded regions, the proposed method produces more complete and accurate detection results. In addition, the Grad-CAM visualizations show that FDA_YOLOv8 focuses more effectively on salient object regions while suppressing irrelevant background interference. This improvement can be attributed to the multi-scale feature aggregation and attention-based feature enhancement introduced in the proposed model, which improve feature representation and discrimination for UAV small-object detection. Overall, these visual results further support the quantitative improvements of FDA_YOLOv8 and demonstrate its effectiveness in UAV-based small object detection scenarios.

## Conclusion

This study demonstrates the effectiveness of FDA_YOLOv8 for small object detection in UAV imagery, leveraging architectural improvements to enhance feature extraction and computational efficiency. The proposed modifications, including a four-detection-head structure, dynamic feature fusion, and lightweight backbone optimization, contribute to improved detection of small objects in complex environments. Experimental results on VisDrone2019 confirm that FDA_YOLOv8 achieves a 5.2% mAP improvement over YOLOv8s while remaining lightweight. Nevertheless, challenges persist in handling extreme clutter and severe occlusions. Future work will focus on enhancing robustness in dense scenes, improving generalization across diverse UAV datasets, and exploring adaptive training strategies for real-time aerial detection.

## Data Availability

The datasets generated during the current study are too large to be publicly shared, but are available from the corresponding author on reasonable request.

## References

[1] Bisio, I., Garibotto, C., Haleem, H., Lavagetto, F. & Sciarrone, A. Traffic analysis through deep-learning-based image segmentation from UAV streaming. *IEEE Internet Things J.* **10**, 6059–6073 (2023).

[2] Chen, Z., Ji, H., Zhang, Y., Zhu, Z. & Li, Y. High-resolution feature pyramid network for small object detection on drone view. *IEEE Transac. Circuits Syst. Video Technol.* 475–489 (2024).

[3] Zou, Z., Chen, K., Shi, Z., Guo, Y. & Ye, J. Object detection in 20 years: A survey. *Proc. IEEE* **11**, 257–276 (2023).

[4] Wei, W., Cheng, Y., He, J. & Zhu, X. A review of small object detection based on deep learning. *Neural Comput. Appl.* **36**, 6283–6303 (2024).

[5] Mahaur, B., Mishra, K. & Kumar, A. An improved lightweight small object detection framework applied to real-time autonomous driving. *Expert Syst. Appl.* **234**, 121036 (2023).

[6] Dalal, N. & Trigg, B. Histograms of oriented gradients for human detection. In *2005 IEEE Conference on Computer Vision and Pattern Recognition (CVPR’05)*, 886–893 (2005).

[7] Agarwal, K. *et al.* Performance analysis of yolov7 and yolov8 models for drone detection. In *2023 International Conference on Network, Multimedia and Information Technology (NMITCON)*, 1–10 (2023).

[8] Zhai, X., Huang, Z., Li, T., Liu, H. & Wang, S. Yolo-drone: An optimized yolov8 network for tiny UAV object detection. *Electronics* **12**, 1–21 (2023).

[9] Ma, C. et al. Yolo-UAV: Object detection method of unmanned aerial vehicle imagery based on efficient multi-scale feature fusion. *IEEE Access* **11**, 126857–126878 (2023).

[10] Wang, G. et al. Uav-yolov8: A small-object-detection model based on improved yolov8 for UAV aerial photography scenarios. *Sensors* **23**, 7190 (2023). 37631727 10.3390/s23167190 PMC10458807

[11] Girshick, R., Donahue, J., Darrell, T. & Malik, J. Rich feature hierarchies for accurate object detection and semantic segmentation. In *Proceedings of the IEEE Conference on Computer Vision and Pattern Recognition*, 580–587 (2014).

[12] Girshick, R. Fast r-cnn. In *Proceedings of the IEEE International Conference on Computer Vision*, 1440–1448 (2015).

[13] Liu, W. *et al.* Ssd: Single shot multibox detector. In *Computer Vision–ECCV 2016: 14th European Conference, Amsterdam, The Netherlands, October 11–14, 2016, Proceedings, Part I 14*, 21–37 (Springer) (2016).

[14] Dai, X. *et al.* Dynamic head: Unifying object detection heads with attentions. In *Proceedings of the IEEE/CVF Conference on Computer Vision and Pattern Recognition*, 7373–7382 (2021).

[15] Li, Y., Yang, X., Tang, J., Xu, Y. & Zhang, S. Deep learning for UAV-based object detection: A survey. *ACM Comput. Surv.* **56**, 1–38 (2023).

[16] Lin, Z., Wang, Y., Zhang, J. & Chu, X. Dynamicdet: A unified dynamic architecture for object detection. In *Proceedings of the IEEE/CVF Conference on Computer Vision and Pattern Recognition (CVPR)*, 21721–21730 (2023).

[17] Jin, X. et al. Delving deep into spatial pooling for squeeze-and-excitation networks. *Pattern Recogn.* **121**, 108159 (2022).

[18] Ouyang, D. *et al.* Efficient multi-scale attention module with cross-spatial learning. In *ICASSP 2023-2023 IEEE International Conference on Acoustics, Speech and Signal Processing (ICASSP)*, 1–5 (IEEE)(2023).

[19] Chen, J. *et al.* Run, don’t walk: Chasing higher flops for faster neural networks. In *Proceedings of the IEEE/CVF Conference on Computer Vision and Pattern Recognition*, 12021–12031 (2023).

[20] Zhu, P. et al. Detection and tracking meet drones challenge. *IEEE Trans. Pattern Anal. Mach. Intell.* **44**, 7380–7399 (2021). 10.1109/TPAMI.2021.3119563 34648430

[21] Mahaur, B. & Mishra, K. Small-object detection based on yolov5 in autonomous driving systems. *Pattern Recogn. Lett.* **168**, 115–122 (2023).

[22] Wang, C.-Y., Bochkovskiy, A. & Liao, H.-Y. M. Yolov7: Trainable bag-of-freebies sets new state-of-the-art for real-time object detectors. In *Proceedings of the IEEE/CVF conference on computer vision and pattern recognition*, 7464–7475 (2023).

[23] Ni, J., Zhu, S., Tang, G., Ke, C. & Wang, T. A small-object detection model based on improved yolov8s for UAV image scenarios. *Remote Sensing* **16**, 2465 (2024).

[24] Chandrashekhar, A., Satyanarayana, B., Gorrepati, R. R., Vasanthi, P. & Prasanna, K. L. An efficient yolov12-based framework for detecting extremely small-scale objects. *Sci. Rep.* **16**, 2062 (2025). 41388062 10.1038/s41598-025-31803-7 PMC12808633

[25] Keshk, H. & Abdallah, A. Real-time UAV-based oil pipeline and visual anomaly detection using yolov26n: A dataset and edge-deployment study. *Drones* **10**, 255 (2026).

[26] Selvaraju, R. R. *et al.* Grad-cam: Visual explanations from deep networks via gradient-based localization. In *Proceedings of the IEEE International Conference on Computer Vision*, 618–626 (2017).

